# Association of N6-methyladenosine readers' genes variation and expression level with pulmonary tuberculosis

**DOI:** 10.3389/fpubh.2022.925303

**Published:** 2022-08-22

**Authors:** Hong-Miao Li, Fei Tang, Li-Jun Wang, Qian Huang, Hai-Feng Pan, Tian-Ping Zhang

**Affiliations:** ^1^Department of Epidemiology and Biostatistics, School of Public Health, Anhui Medical University, Hefei, China; ^2^Department of Infectious Diseases, The First Affiliated Hospital of Anhui Medical University, Hefei, China; ^3^Department of Interventional Pulmonology and Endoscopic Diagnosis and Treatment Center, Anhui Chest Hospital, Hefei, China; ^4^Department of Public Health, Medical Department, Qinghai University, Xining, China; ^5^Department of Scientific Research, The First Affiliated Hospital of USTC, Division of Life Sciences and Medicine, University of Science and Technology of China, Hefei, China

**Keywords:** pulmonary tuberculosis, N6-methyladenosine, single-nucleotide polymorphisms, epidemiology, infectious diseases

## Abstract

N6-Methyladenosine (m6A) is associated with many biological processes and the development of multiple diseases. The aim of this study was to analyze the association of m6A readers' genes variation, as well as their expression levels, with pulmonary tuberculosis (PTB). A total of 11 single-nucleotide polymorphisms (SNPs) in m6A readers' genes (i.e., *YTHDF1* rs6122103, rs6011668, *YTHDF2* rs602345, rs3738067, *YTHDF3* rs7464, rs12549833, *YTHDC1* rs3813832, rs17592288, rs2293596, and *YTHDC2* rs6594732, and rs2416282) were genotyped by SNPscan™ technique in 457 patients with PTB and 466 normal controls. The m6A readers' genes expression levels in peripheral blood mononuclear cells (PBMCs) from 78 patients with PTB and 86 normal controls were detected by quantitative real-time reverse transcription polymerase chain reaction (qRT-PCR). There was no significant association between all SNPs in *YTHDF1, YTHDF2, YTHDF3, YTHDC1*, and *YTHDC2* genes and PTB susceptibility. The increased frequencies of *YTHDF2* rs3738067 GG genotype and *YTHDC1* rs3813832 CC genotype, C allele, were, respectively, found in PTB patients with hypoproteinemia and fever. *YTHDC2* rs6594732 variant was significantly associated with drug-induced liver damage and sputum smear-positive, and the rs2416282 variant was significantly associated with fever in patients with PTB. Compared with controls, the YTHDF1, YTHDF2, YTHDF3, YTHDC1, and YTHDC2 mRNA levels were significantly decreased in PTB. Moreover, YTHDF1 level was negatively associated with erythrocyte sedimentation rate (ESR), and YTHDF3 and YTHDC1 levels were negatively related to alanine aminotransferase (ALT) in patients with PTB. Our results demonstrated that *YTHDF1, YTHDF2, YTHDF3, YTHDC1*, and *YTHDC2* genes SNPs did not contribute to PTB susceptibility, while their decreased levels in patients with PTB suggested that these m6A readers might play significant roles in PTB.

## Introduction

Tuberculosis (TB), caused by *Mycobacterium tuberculosis* (MTB), is a serious infectious disease with high morbidity and mortality. Pulmonary TB (PTB) is the most common and poses a serious threat to public health. China is still the third highest burdened country and accounts for 8.4% of the total global patients in 2019 (1). Studies had shown that the occurrence and development of TB was mainly determined by a complex interaction between multiple factors, including MTB strains and environmental and host genetic factors (2–4). Host genetics was confirmed to play an important role in determining disease progression and prognosis after MTB infection; hence, identifying the factors that influence disease susceptibility could provide important evidence for the design of effective control strategies. A considerable number of genetic variants for PTB susceptibility had been identified, while it could only account for part of the heritability of PTB (5–7).

Epigenetic modification also played an important role in the pathogenesis of PTB, and DNA methylation was an important epigenetic marker for the risk of several diseases (8, 9). Both cytosine and adenine could be methylated in DNA, resulting in N4-methylcytosine, 5-methylcytosine, and N6-methyladenosine (m6A) (10). m6A had critical modification effects on a variety of cytological processes, including nuclear export, splicing, translatability, and stability of mRNA, and was closely related to the pathogenesis of various diseases (11). Moreover, m6A methylation was found in the *MTB* genome, and DNA methylation could regulate the expression of genes related to the hypoxia survival of *MTB*; hence, m6A methylation was likely to be involved in the pathogenesis of PTB (12, 13). Disease-related genetic variations had been proved to influence m6A methylation by altering the RNA sequence of its target sites or key flanking nucleotides, suggesting that m6A-related single-nucleotide polymorphisms (SNPs) might influence the stability of mRNA, which might contribute to the development of human disease (14). Some studies had explored the potential association between genetic variation in the m6A-modified core genes and the risk of human diseases (15, 16).

N6-Methyladenosine-associated RNA binding proteins (readers), including YTH m6A RNA-binding protein 1 (YTHDF1), YTHDF2, YTHDF3, YTH domain-containing 1 (YTHDC1), and YTHDC2, played a key role in m6A modification by modulating mRNA fate (17, 18). Moreover, functional SNPs in *YTHDF1* might influence its expression and binding ability to m6A-modified RNA, which eventually affect tumorgenesis (19). However, the role of these m6A readers in PTB was still unclear. Thus, we performed this study to evaluate the associations of m6A readers' (YTHDF1, YTHDF2, YTHDF3, YTHDC1, and YTHDC2) gene variation and their expression levels with PTB susceptibility in a Chinese Han population.

## Materials and methods

### Study participants

In this study, we consecutively recruited a total of 923 subjects including 457 patients with PTB and 466 normal controls to analyze the association between *YTHDF1, YTHDF2, YTHDF3, YTHDC1*, and *YTHDC2* genes polymorphisms and PTB susceptibility. Then, 78 patients with PTB and 86 normal controls were enrolled to detect these genes levels. All patients with PTB were selected from the Department of Tuberculosis at Anhui Chest Hospital, and diagnosed by a specialist on the basis of these criteria as follows: suspicious clinical symptoms, chest radiography, sputum and/or bronchoalveolar lavage fluid MTB culture, microscopy for acid-fast bacilli, and effect of anti-TB treatment. The exclusion criteria for patients with PTB included HIV-positive, hepatitis, malignancy, and immune-compromised conditions. The normal controls were enrolled from health examination center in the same area and needed to be asymptomatic with sputum smear- and culture-negative, normal chest radiograph, and no history of TB. All patients with PTB and normal controls were the Chinese Han population, and no biological relationship was existed in these study subjects.

This study was approved by the Medical Ethics Committee of Anhui Medical University (20200250), and written informed consent was obtained from all subjects prior to the study. Then, the peripheral blood samples, demographic characteristics, clinical manifestations, and laboratory indicators were collected from study participants. The clinical manifestations of patients with PTB included fever, drug resistance, drug-induced liver injury (DILI), pulmonary infection, leukopenia, and sputum smears, and the laboratory indicators of patients with PTB included erythrocyte sedimentation rate (ESR), total bilirubin (TBIL), aspartate aminotransferase (AST), and alanine aminotransferase (ALT).

### SNP selection, DNA extraction, and genotyping

In this study, we screened several specific tagSNPs in each gene for genotyping. The tagSNPs were selected with a minor allele frequency (MAF) ≥ 0.05 in CHB, capturing all the common SNPs located in the chromosome locations of these m6A readers (*YTHDF1, YTHDF2, YTHDF3, YTHDC1*, and *YTHDC2*) and their flanking 2.0 kbp region by using genetic data of CHB from Ensembl genome browser 85 and CHBS_1000g. The selection was conducted using the linkage disequilibrium (LD) analysis according to *r*^2^ threshold > 0.8, and the Haploview 4.0 software (Cambridge, MA, USA). We also reviewed the existing studies regarding the association of these gene polymorphisms with disease susceptibility, and searched other potentially functional SNPs. Finally, we selected two tagSNPs (rs6122103 and rs6011668) in *YTHDF1*, two tagSNPs (rs602345 and rs3738067) in *YTHDF2*, two tagSNPs (rs7464 and rs12549833) in *YTHDF3*, three tagSNPs (rs3813832, rs17592288, and rs2293596) in *YTHDC1*, and two tagSNPs (rs6594732 and rs2416282) in *YTHDC2* for genotyping.

The genomic DNA was extracted from the peripheral blood leukocytes by the Flexi Gene-DNA Kit (Qiagen, Valencia, CA). The SNPscan™ technique, with technical support from the Center for Genetic & Genomic Analysis, Genesky Biotechnologies Inc. (Shanghai), was used for genotyping. Those individuals with a 100% genotyping success rate for the above SNPs were included in the final analysis.

### Quantitative real-time reverse transcription polymerase chain reaction (qRT-PCR)

The PBMCs were isolated from 5 ml peripheral blood and stored at −80°C until processed. Total RNA was extracted from PBMCs using TRIzol Reagent (Invitrogen, Carlsbad, CA, USA), and the RNA concentration was detected with NanoDrop 2000 spectrophotometer (Thermo Scientific, USA). Next, the total RNA was reversely transcribed into cDNA by the PrimeScriptTM RT Reagent Kit (Takara Bio Inc., Japan).

In this study, the YTHDF1, YTHDF2, YTHDF3, YTHDC1, and YTHDC2 expression levels in PBMC were measured by quantitative real-time reverse transcription polymerase chain reaction (qRT-PCR) with SYBR Green (SYBR Premix Ex Taq II, Takara Bio Inc., Japan), and this experiment was carried out in duplicate by using QuantStudio 12K Flex Real-Time PCR system (Applied Biosystems, Foster City, CA, USA). Thermal cycling conditions were as follows: 95°C for 1 min, followed by 42 cycles at 95°C for 10 s, 60°C for 30 s, and 72°C for 1 min. The relative expression levels of these genes were calculated by using the 2^−ΔΔCt^ method normalized to an endogenous control, and the housekeeping gene β-actin was used as an internal control in the same sample.

### Statistical analysis

All the statistical analysis was performed using SPSS version 23.0 and data were shown as frequency, percentage, mean ± standard deviation (SD), and median (quartile range) according to their types. The differences in these m6A readers' expression levels between two groups and three groups were, respectively, analyzed by the Mann–Whitney *U* test and Kruskal–Wallis *H* test, and correlation analysis was performed with Spearman's rank correlation coefficient test. We performed the Hardy–Weinberg equilibrium test in normal controls using chi-square (χ^2^). The difference in each SNP genotype and allele frequency distribution between different groups was evaluated with χ^2^; odds ratio (OR) and 95% confidence interval (CI) were determined by the logistic regression analysis. Two genetic models (dominant model and recessive model) were used for statistical analysis, and haplotype analysis was conducted using the SHEsis software (20). A *P*-value of < 0.05 was considered statistically significant.

## Results

In the genotyping experiment, 457 patients with PTB consisted of 264 men and 193 women, with a mean age of 45.42 ± 17.74 years, and 466 controls consisted of 202 men and 264 women, with an average age of 43.43 ± 12.95 years (Supplementary Table S1). In the qRT-PCR experiment, the PTB group enrolled 51 men and 27 women, with a mean age of 49.83 ± 18.59 years, and 57 men and 29 women were included in the control group, with an average age of 48.47 ± 17.40 years (Supplementary Table S1).

### Association of m6A readers' genes polymorphisms with the susceptibility for PTB

The allele and genotype frequencies of all SNPs in *YTHDF1, YTHDF2, YTHDF3, YTHDC1*, and *YTHDC2* genes are shown in Table 1, and the genotype distribution of all SNPs in controls was conformed to the Hardy–Weinberg equilibrium. There were no significant differences in allele and genotype distributions of *YTHDF11* rs6122103 and rs6011668 polymorphism between patients with PTB and controls (all *P-*values > 0.05). Similarly, we did not find any significant association between *YTHDF2* rs602345, rs3738067, *YTHDF3* rs7464, rs12549833, *YTHDC1* rs3813832, rs17592288, rs2293596, *YTHDC2* rs6594732, and rs2416282 polymorphisms and the risk of PTB. The association between these SNPs and PTB susceptibility under the dominant model and recessive model was also analyzed; however, no significant association was detected.

**Table 1:** Genotype and allele frequencies of m6A readers' genes in patients with PTB and normal controls.

**SNP**	**Analyze model**		**PTB patients**	**Controls**	***P*-value**	**OR (95 % CI)**
**YTHDF1**						
rs6122103	Genotype	GG	214 (46.83)	201 (43.13)	Reference	
		GA	191 (41.79)	213 (45.71)	0.220	0.842 (0.640, 1.108)
		AA	52 (11.38)	52 (11.16)	0.775	0.939 (0.611, 1.444)
	Allele	G	619 (67.72)	615 (65.99)	Reference	
		A	295 (32.28)	317 (34.01)	0.428	0.949 (0.834, 1.080)
	Dominant model	GA+AA	243 (53.17)	265 (56.87)	Reference	
		GG	214 (46.83)	201 (43.13)	0.428	0.949 (0.834, 1.080)
	Recessive model	GA+GG	405 (88.62)	414 (88.84)	Reference	
		AA	52 (11.38)	52 (11.16)	0.428	0.949 (0.834, 1.080)
rs6011668	Genotype	CC	323 (70.68)	323 (69.31)	Reference	
		TC	127 (27.79)	132 (28.33)	0.793	0.962 (0.721, 1.284)
		TT	7 (1.53)	11 (2.36)	0.356	0.636 (0.244, 1.662)
	Allele	C	773 (84.57)	778 (83.48)	Reference	
		T	141 (15.43)	154 (16.52)	0.520	0.934 (0.757, 1.151)
	Dominant model	CC	323 (70.68)	323 (69.31)	0.651	1.020 (0.937, 1.110)
		TC+TT	134 (29.32)	143 (30.69)	Reference	
	Recessive model	TC+CC	450 (98.47)	455 (97.64)	Reference	
		TT	7 (1.53)	11 (2.36)	0.363	0.649 (0.254, 1.659)
**YTHDF2**						
rs602345	Genotype	CC	327 (71.55)	337 (72.32)	Reference	
		TC	114 (24.95)	117 (25.11)	0.978	1.004 (0.744, 1.355)
		TT	16 (3.50)	12 (2.58)	0.632	1.375 (0.640, 2.949)
	Allele	C	768 (84.03)	791 (84.87)	Reference	
		T	146 (15.97)	141 (15.13)	0.616	1.056 (0.854, 1.306)
	Dominant model	CC	327 (71.55)	337 (72.32)	0.796	0.989 (0.913, 1.072)
		TC+TT	130 (28.45)	129 (27.68)	Reference	
	Recessive model	TC+CC	441 (96.50)	454 (97.42)	Reference	
		TT	16 (3.50)	12 (2.58)	0.412	1.360 (0.650, 2.842)
rs3738067	Genotype	AA	254 (55.58)	259 (55.58)	Reference	
		GA	166 (36.32)	171 (36.70)	0.942	0.990 (0.752, 1.303)
		GG	37 (8.10)	36 (7.73)	0.851	1.048 (0.642, 1.711)
	Allele	A	674 (73.74)	689 (73.93)	Reference	
		G	240 (26.26)	243 (26.07)	0.928	1.007 (0.864, 1.174)
	Dominant model	AA	254 (55.58)	259 (55.58)	1.000	1.000 (0.891, 1.122)
		GA+GG	203 (44.42)	207 (44.42)	Reference	
	Recessive model	GA+AA	420 (91.90)	430 (92.27)	Reference	
		GG	37 (8.10)	36 (7.73)	0.835	1.048 (0.675, 1.628)
**YTHDF3**						
rs7464	Genotype	AA	249 (54.49)	246 (52.79)	Reference	
		GA	176 (38.51)	182 (39.06)	0.742	0.955 (0.728, 1.254)
		GG	32 (7.00)	38 (8.15)	0.473	0.832 (0.504, 1.375)
	Allele	A	674 (73.74)	674 (72.32)	Reference	
		G	240 (26.26)	258 (27.68)	0.491	0.949 (0.816, 1.102)
	Dominant model	AA	249 (54.49)	246 (52.79)	0.605	1.032 (0.915, 1.164)
		GA+GG	208 (45.51)	220 (47.21)	Reference	
	Recessive model	GA+AA	425 (93.00)	428 (91.85)	Reference	
		GG	32 (7.00)	38 (8.15)	0.509	0.859 (0.546, 1.350)
rs12549833	Genotype	AA	199 (43.54)	192 (41.20)	Reference	
		AG	202 (44.20)	218 (46.78)	0.426	0.894 (0.679, 1.178)
		GG	56 (12.25)	56 (12.02)	0.867	0.965 (0.634, 1.469)
	Allele	A	600 (65.65)	602 (64.59)	Reference	
		G	314 (34.35)	330 (35.41)	0.635	0.970 (0.857, 1.099)
	Dominant model	AG+GG	258 (56.46)	274 (58.80)	Reference	
		AA	199 (43.54)	192 (41.20)	0.471	1.057 (0.909, 1.229)
	Recessive model	AG+AA	401 (87.75)	410 (87.98)	Reference	
		GG	56 (12.25)	56 (12.02)	0.912	0.997 (0.951, 1.046)
**YTHDC1**						
rs3813832	Genotype	TT	236 (51.64)	237 (50.86)	Reference	
		TC	190 (41.58)	197 (42.27)	0.816	0.969 (0.740, 1.267)
		CC	31 (6.78)	32 (6.87)	0.918	0.973 (0.575, 1.646)
	Allele	T	662 (72.43)	671 (72.00)	Reference	
		C	252 (27.57)	261 (28.00)	0.835	0.983 (0.850, 1.141)
	Dominant model	TT	236 (51.64)	237 (50.86)	0.812	1.015 (0.895, 1.152)
		TC+CC	221 (48.36)	229 (49.14)	Reference	
	Recessive model	TC+TT	426 (93.22)	434 (93.13)	Reference	
		CC	31 (6.78)	32 (6.87)	0.960	0.988 (0.613, 1.591)
rs17592288	Genotype	AA	417 (91.25)	432 (92.70)	Reference	
		AC	39 (8.53)	34 (7.30)	0.480	1.188 (0.736, 1.919)
		CC	1 (0.22)	0 (0)	1.000	—
	Allele	A	873 (95.51)	898 (96.35)	Reference	
		C	41 (4.49)	34 (3.65)	0.362	0.991 (0.973, 1.010)
	Dominant model	AA	417 (91.25)	432 (92.70)	0.415	1.200 (0.774, 1.860)
		AC+CC	40 (8.75)	34 (7.30)	Reference	
	Recessive model	AC+AA	456 (99.78)	466 (100.00)	Reference	
		CC	1 (0.22)	0 (0)	0.312	0.998 (0.994, 1.002)
rs2293596	Genotype	TT	300 (65.65)	309 (66.31)	Reference	
		TC	138 (30.2)	140 (30.04)	0.917	1.015 (0.764, 1.348)
		CC	19 (4.16)	17 (3.65)	0.682	1.151 (0.587, 2.257)
	Allele	T	738 (80.74)	758 (81.33)	Reference	
		C	176 (19.26)	174 (18.67)	0.748	1.031 (0.854, 1.246)
	Dominant model	TT	300 (65.65)	309 (66.31)	0.832	0.990 (0.902, 1.086)
		TC+CC	157 (34.35)	157 (33.69)	Reference	
	Recessive model	TC+TT	438 (95.84)	449 (96.35)	Reference	
		CC	19 (4.16)	17 (3.65)	0.689	1.140 (0.600, 2.165)
**YTHDC2**						
rs6594732	Genotype	CC	302 (66.08)	281 (60.30)	Reference	
		CA	137 (29.98)	166 (35.62)	0.063	0.768 (0.581, 1.015)
		AA	18 (3.94)	19 (4.08)	0.710	0.881 (0.453, 1.714)
	Allele	C	741 (81.07)	728 (78.11)	Reference	
		A	173 (18.93)	204 (21.89)	0.115	0.865 (0.722, 1.036)
	Dominant model	CC	302 (66.08)	281 (60.30)	0.069	1.096 (0.993, 1.210)
		CA+AA	155 (33.92)	185 (39.70)	Reference	
	Recessive model	CA+CC	439 (96.06)	447 (95.92)	Reference	
		AA	18 (3.94)	19 (4.08)	0.915	0.966 (0.514, 1.817)
rs2416282	Genotype	AA	147 (32.17)	144 (30.90)	Reference	
		CA	217 (47.48)	246 (52.79)	0.329	0.864 (0.644, 1.159)
		CC	93 (20.35)	76 (16.31)	0.350	1.199 (0.819, 1.753)
	Allele	A	511 (55.91)	534 (57.30)	Reference	
		C	403 (44.09)	398 (42.70)	0.547	1.033 (0.930, 1.146)
	Dominant model	CA+CC	310 (67.83)	322 (69.10)	Reference	
		AA	147 (32.17)	144 (30.90)	0.679	0.982 (0.899, 1.072)
	Recessive model	CA+AA	364 (79.65)	390 (83.69)	Reference	
		CC	93 (20.35)	76 (16.31)	0.112	1.248 (0.949, 1.641)

The results regarding the association between these SNPs and several clinical manifestations in patients with PTB suggested that *YTHDF2* rs3738067 GG genotype frequency was significantly increased in PTB patients with hypoproteinemia when compared to the patients without hypoproteinemia (*P* = 0.009), and *YTHDC1* rs3813832 CC genotype, C allele, was significantly related to the occurrence of fever in patients with PTB (*P* = 0.044 and *P* = 0.027, respectively) (Supplementary Table S2, Table 2). In the *YTHDC2* gene, the elevated frequencies of rs6594732 AA genotype and A allele were significantly associated with DILI in patients with PTB (*P* = 0.033 and *P* = 0.008, respectively), while the decreased frequencies of rs6594732 AA genotype and A allele were significantly associated with sputum smear-positive (*P* = 0.039 and *P* = 0.020, respectively). In addition, rs2416282 CC genotype frequency was significantly decreased in PTB patients with fever (*P* = 0.013).

**Table 2:** The positive findings of associations between m6A readers' genes polymorphisms and clinical features of patients with PTB.

**SNP**	**Allele**	**Clinical features**	**Group**	**Genotype**			***P*-value**	**Allele**		***P*-value**
	**(M/m)**			**MM**	**Mm**	**mm**		**M**	**m**	
YTHDF2 rs3738067	A/G	Hypoproteinemia	+	39	9	7	0.009	87	23	0.216
			-	265	177	38		707	253	
YTHDC1 rs3813832	T/C	Fever	+	27	38	6	0.044	92	50	0.027
			-	209	152	25		570	202	
YTHDC2 rs6594732	C/A	DILI	+	39	29	6	0.033	107	41	0.008
			-	306	139	16		751	171	
YTHDC2 rs6594732	C/A	Sputum smear	+	106	44	2	0.039	256	48	0.020
			-	211	113	20		535	153	
YTHDC2 rs2416282	A/C	Fever	+	15	45	11	0.013	75	67	0.420
			-	132	172	82		436	336	

+, with; -, without.

### Haplotype analysis

The haplotype of *YTHDF1, YTHDF2, YTHDF3, YTHDC1*, and *YTHDC2* genes was detected using the SHEsis software, and then the differences in these haplotype frequencies between patients with PTB and controls were compared. Three main haplotypes each (AC, GC, GT), (CA, CG, TG), (AA, AG, GA), and (AC, CA, CC) for YTHDF2, YTHDF1, YTHDF2, and YTHDF2, respectively, and four main haplotypes (CAT, TAC, TAT, TCT) for YTHDC1 were detected.

As shown in Table 3, we found that *YTHDC2* gene CC haplotype frequency was significantly higher in patients with PTB than in controls (*P* = 0.033). Meanwhile, the frequencies of other haplotypes were not statistically associated with PTB susceptibility.

**Table 3:** Haplotype analysis of m6A readers' genes in patients with PTB and controls.

**Haplotype**	**PTB patients**	**Controls**	***P*-value**	**OR (95% CI)**
**YTHDF1 rs6122103-rs6011668**				
AC	294.95 (32.3)	316.97 (34.0)	0.427	0.924 (0.762,1.122)
GC	478.05 (52.3)	461.03 (49.5)	0.223	1.120 (0.933,1.345)
GT	140.95 (15.4)	153.97 (16.5)	0.519	0.921 (0.718,1.182)
**YTHDF2 rs602345-rs3738067**				
CA	672.90 (73.6)	689.00 (73.9)	0.916	0.989 (0.803,1.217)
CG	95.10 (10.4)	102.00 (10.9)	0.714	0.946 (0.704,1.272)
TG	144.90 (15.9)	141.00 (15.1)	0.659	1.058 (0.822,1.362)
**YTHDF3 rs7464-rs12549833**				
AA	360.02 (39.4)	344.02 (36.9)	0.273	1.111 (0.920,1.340)
AG	313.98 (34.4)	329.98 (35.4)	0.635	0.955 (0.788,1.156)
GA	239.98 (26.3)	257.98 (27.7)	0.491	0.930 (0.757,1.143)
**YTHDC1 rs3813832- rs17592288- rs2293596**				
CAT	251.96 (27.6)	260.97 (28.0)	0.835	0.979 (0.798,1.200)
TAC	175.96 (19.3)	173.96 (18.7)	0.748	1.039 (0.823,1.311)
TAT	445.04 (48.7)	463.04 (49.7)	0.671	0.961 (0.801,1.154)
TCT	40.99 (4.5)	33.99 (3.6)	0.362	1.240 (0.780,1.973)
**YTHDC2 rs6594732- rs2416282**				
AC	172.99 (18.9)	202.70 (21.7)	0.128	0.838 (0.668,1.052)
CA	510.99 (55.9)	532.70 (57.2)	0.565	0.947 (0.788,1.139)
CC	230.01 (25.2)	195.30 (21.0)	0.033	1.266 (1.019,1.573)

frequency < 0.03 in both controls & PTB patients has been dropped.

Bold value means P < 0.05.

### m6A readers' expression levels in patients with PTB and normal controls

**Figure 1:**
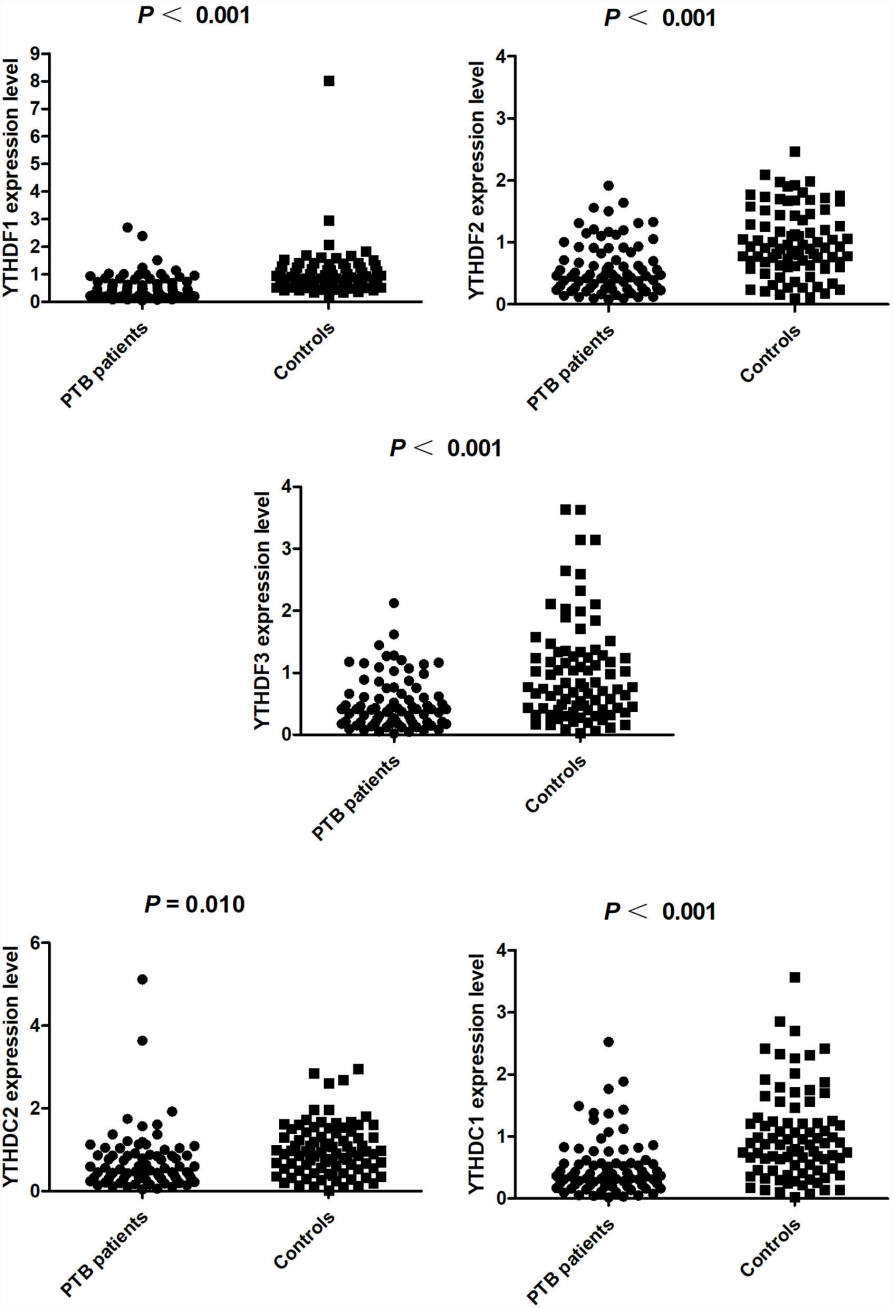
The m6A readers' expression levels in patients with PTB and normal controls.

The mRNA expression levels of YTHDF1, YTHDF2, YTHDF3, YTHDC1, and YTHDC2 in PBMCs from patients with PTB and normal controls by qRT-PCR were further detected. As shown in Figure 1, the YTHDF1, YTHDF2, YTHDF3, YTHDC1, and YTHDC2 expression levels in patients with PTB were significantly lower than that in normal controls (all *P-*values < 0.05).

The correlation of these m6A readers' expression levels with several common clinical features of patients with PTB was also analyzed. The results showed that YTHDF1, YTHDF2, YTHDF3, YTHDC1, and YTHDC2 mRNA levels were not associated with the occurrence of those clinical features, including fever, DILI, pulmonary infection, hypoproteinemia, leukopenia, sputum smear-positive, in patients with PTB (Supplementary Table S2). In addition, the expression level of YTHDF1 was negatively associated with ESR in patients with PTB (*P* = 0.039), and YTHDF3 and YTHDC1 levels were negatively associated with ALT (*P* = 0.031 and *P* = 0.012, respectively). However, there were no significant correlations of these m6A reader levels with TBIL and AST of patients with PTB (Table 4).

**Table 4:** The correlation between m6A readers' genes expression levels and ESR, TBIL, ALT, and AST of patients with PTB.

**Clinical parameters**	**YTHDF1 level**		**YTHDF2 level**		**YTHDF3 level**		**YTHDC1 level**		**YTHDC2 level**	
	**r_s_**	***P*–value**	**r_s_**	***P*–value**	**r_s_**	***P*–value**	**r_s_**	***P*–value**	**r_s_**	***P*–value**
ESR	−0.238	0.039	−0.104	0.373	−0.031	0.788	−0.120	0.303	−0.127	0.274
TBIL	−0.050	0.671	−0.011	0.922	0.101	0.383	0.109	0.348	0.103	0.378
ALT	−0.011	0.923	−0.172	0.134	−0.245	0.031	−0.285	0.012	−0.175	0.128
AST	−0.040	0.729	−0.184	0.111	−0.153	0.187	−0.184	0.112	−0.158	0.173

r_s:_Spearman's rank correlation coefficient.

### Associations between m6A readers' genes polymorphisms with their levels in patients with PTB

A total of 62 patients with PTB were included to analyze the associations between these m6A readers' genes variation and their expression levels. The results demonstrated that there might be some differences in the expression levels of these genes among different genotypes, but no difference reached a statistical significance (all *P*-values > 0.05) (Supplementary Table S3).

## Discussion

RNA m6A modification was considered the most abundant, pervasive, and important chemical modification in eukaryotic RNAs and could affect all aspects of RNA metabolism, such as RNA transcription, processing, translation, and transportation (21, 22). The m6A modification process was accomplished by a series of proteins, which were mainly divided into “writers,” “erasers,” and “readers,” according to their different roles (18). Due to the important role of RNA m6A modification in a variety of biological processes, it was reasonable to believe that genetic variation in m6A modified genes had been involved in the development of multiple human diseases. For example, genetic variations in m6A modification core genes had been shown to be associated with cancer susceptibility in many studies (16, 23). Recently, the association between m6A modification genes variation and PTB susceptibility had also been discussed. Previous studies have found that rs9939609 polymorphism in fat mass and obesity-associated protein (*FTO*), known as m6A demethylases, was associated with the risk of PTB (24, 25). However, research on m6A critical gene SNPs on PTB risk was still at the primary stage. Therefore, we focused on the relationship between five m6A readers genes (*YTHDF1, YTHDF2, YTHDF3, YTHDC1*, and *YTHDC2*) SNPs and expression levels with PTB in this study.

The final consequences of m6A modification on mRNA fate were executed by “reader” proteins, and these readers' genes mainly included the YTH family (YTHDC1-2 and YTHDF1-3). The contribution of m6A readers' genes to cancer was widely studied, and multiple SNPs located in m6A readers' genes had been found to affect the risk of cancer (23, 26, 27). Liu *et al*. found that compared with the CC genotype, *YTHDF1* rs6011668 CT/TT genotype was associated with an increased risk of Wilms tumor in patients ≤18 months in stratification analysis, although this SNP might not contribute to the risk of Wilms tumor (26). The results by Zeng *et al*. demonstrated that *YTHDF2* rs3738067 A>G could decrease neuroblastoma risk in the Chinese children (27). First, we analyzed the association between *YTHDF1* rs6122103, rs6011668, *YTHDF2* rs602345, rs3738067, *YTHDF3* rs7464, and rs12549833 polymorphism and PTB susceptibility; however, no statistically significant findings were observed. In this sense, this viewpoint was providing evidence that these gene polymorphisms might not affect the susceptibility for PTB. In the progression of PTB, patients were usually accompanied by multiple complications and clinical manifestations, including fever, pulmonary infection, and drug resistance, which were also affected by genetic variation (6, 28). In this study, our results demonstrated that the *YTHDF2* rs3738067 GG genotype was significantly associated with the occurrence of hypoproteinemia in PTB. It was worth noting that the *YTHDF2* rs3738067 variant might be involved in the development of PTB, and this result would be verified and further explored in our future research.

Some studies have analyzed the relationship between *YTHDC1, YTHDC2* gene variation, and cancer susceptibility. *YTHDC1* rs3813832 TC genotype significantly reduced the susceptibility of neuroblastoma, and rs2293596 T>C polymorphism might contribute to hepatoblastoma susceptibly (29, 30). Another study suggested that the *YTHDC2* rs2416282 variant contributed to esophageal squamous-cell carcinoma risk by regulating YTHDC2 expression (31). Nonetheless, we failed to find any relationships between the selected SNPs in *YTHDC1* (rs3813832, rs17592288, and rs2293596), *YTHDC2* (rs6594732 and rs2416282), and PTB risk in this study. In addition, we found a statistically significant association between CC haplotype in *YTHDC2* and susceptibility to PTB, since haplotypes tended to have a stronger power of predicting disease-related genes than SNP (32). Hence, our results suggested a potential role of *YTHDF2* gene variation in PTB susceptibility, while the specific mechanisms needed to be further explored. Our results also showed that *YTHDC1* rs3813832 and *YTHDC2* rs2416282 variants were significantly related to fever and the *YTHDC2* rs6594732 variant was significantly associated with DILI and sputum smear-positive in patients with PTB. These clinical features could seriously affect the treatment and prognosis of patients with PTB, and the abovementioned SNPs were somewhat used to predispose the occurrence of these clinical features in patients with PTB. Therefore, we speculated that these findings might help to make more appropriate treatment choices for patients with PTB.

Interestingly, increasing evidence had indicated that the expression levels of m6A readers were closely related to the pathogenesis and progression of many diseases. A number of studies had shown that YTHDF1 was overexpressed in various cancers, including colorectal cancer, hepatocellular carcinoma, and breast cancer, and was closely related to the increased risk of these cancers (33–35). Decreased mRNA expression of YTHDF2 was found in patients with systemic lupus erythematosus and rheumatoid arthritis (RA) compared with controls, and YTHDF2 mRNA level in peripheral blood was a risk factor for RA by logistic regression analysis (36, 37). However, few studies had been conducted regarding m6A reader expression levels and the risk of PTB. Our study provided the first evidence that YTHDF1, YTHDF2, YTHDF3, YTHDC1, and YTHDC2 mRNA levels in patients with PTB were significantly decreased than that in controls. This result showed that these m6A readers might be involved in PTB occurrence, and the decreased expression of YTHDF1, YTHDF2, YTHDF3, YTHDC1, and YTHDC2 might be used as auxiliary indicators for PTB diagnosis. Then, we also investigated whether the expressions of these m6A readers in the peripheral blood of patients with PTB could reflect the clinical characteristics of this disease. We showed the expression of peripheral blood YTHDF1 correlated with ESR, and YTHDF3 and YTHDC1 levels were negatively associated with ALT. These findings would help improve our understanding of m6A reader in the development of PTB.

Taken together, we have demonstrated in this study for the first time that *YTHDF1, YTHDF2, YTHDF3, YTHDC1*, and *YTHDC2* genes polymorphisms might not contribute to the susceptibility to PTB. However, several SNPs in *YTHDF2, YTHDC1*, and *YTHDF2* genes were significantly associated with some clinical features in patients with PTB. Moreover, decreased expression levels of YTHDF1, YTHDF2, YTHDF3, YTHDC1, and YTHDC2 in patients with PTB indicated the important roles of these m6A readers in PTB, which might be considered auxiliary biomarkers for PTB diagnosis. It was worth noting that some limitations existed in this study. First, m6A readers' genes variation greatly modified the clinical manifestations of patients with PTB, especially drug resistance, but the mechanism remained unaccounted in this study. Second, this study only found the decreased levels of m6A readers' mRNA in PBMCs from patients with PTB, which should be verified at the protein level. Moreover, this study was only conducted in the Chinese Han population and the influence of m6A readers' genes variation and expression level on PTB needed to be confirmed in other ethnicities. Therefore, functional and replication studies with different ethnic groups and larger sample size were warranted to further explore the exact role of these m6A readers in PTB.

## Data availability statement

The original contributions presented in the study are included in the article/Supplementary files, further inquiries can be directed to the corresponding author.

## Ethics statement

The studies involving human participants were reviewed and approved by the Ethical Committee of Anhui Medical University. The patients/participants provided their written informed consent to participate in this study.

## Author contributions

H-FP and T-PZ designed the study. H-ML and FT conducted the experiment. FT and L-JW participated in the collection of samples. QH performed the statistical analyses. H-ML and T-PZ drafted the manuscript. H-FP contributed to manuscript revision. All the authors approved the final submitted version.

## Funding

This study was supported by grants from the National Natural Science Foundation of China (82003515).

## Conflict of interest

The authors declare that the research was conducted in the absence of any commercial or financial relationships that could be construed as a potential conflict of interest.

## Publisher's note

All claims expressed in this article are solely those of the authors and do not necessarily represent those of their affiliated organizations, or those of the publisher, the editors and the reviewers. Any product that may be evaluated in this article, or claim that may be made by its manufacturer, is not guaranteed or endorsed by the publisher.

## References

[1] World Health Organization. Global Tuberculosis Report 2020. Available online at: https://apps.who.int/iris/bitstream/handle/10665/336069/9789240013131-eng.pdf (accessed April 1, 2022).

[2] O'Garra A Redford PS McNab FW Bloom CI Wilkinson RJ Berry MP. The immune response in tuberculosis. Annu Rev Immunol. (2013) 31:475–527. 10.1146/annurev-immunol-032712-095939 23516984

[3] Fox GJ Redwood L Chang V Ho J. The effectiveness of individual and environmental infection control measures in reducing the transmission of mycobacterium tuberculosis: a systematic review. Clin Infect Dis. (2021) 72:15–26. 10.1093/cid/ciaa719 32502271

[4] Li HM Wang LJ Tang F Pan HF Zhang TP. Association of leptin and leptin receptor genes variants and pulmonary tuberculosis susceptibility, clinical manifestations in a Chinese population. Microb Pathog. (2022) 165:105499. 10.1016/j.micpath.2022.105499 35325792

[5] Varzari A Deyneko IV Tudor E Grallert H Illig T. Synergistic effect of genetic polymorphisms in TLR6 and TLR10 genes on the risk of pulmonary tuberculosis in a Moldavian population. Innate Immun. (2021) 27:365–76. 10.1177/17534259211029996 34275341 PMC8419295

[6] Zhang TP Chen SS Zhang GY Shi SJ Wei L Li HM. Association of vitamin D pathway genes polymorphisms with pulmonary tuberculosis susceptibility in a Chinese population. Genes Nutr. (2021) 16:6. 10.1186/s12263-021-00687-3 33882819 PMC8061222

[7] Zheng R Li Z He F Liu H Chen J Chen J. Genome-wide association study identifies two risk loci for tuberculosis in Han Chinese. Nat Commun. (2018) 9:4072. 10.1038/s41467-018-06539-w 30287856 PMC6172286

[8] Wang M Kong W He B Li Z Song H Shi P. Vitamin D and the promoter methylation of its metabolic pathway genes in association with the risk and prognosis of tuberculosis. Clin Epigenetics. (2018) 10:118. 10.1186/s13148-018-0552-6 30208925 PMC6136159

[9] Chen KD Huang YH Ming-Huey Guo M. The human blood DNA methylome identifies crucial role of beta-catenin in the pathogenesis of Kawasaki disease. Oncotarget. (2018) 9:28337–50. 10.18632/oncotarget.25305 29983864 PMC6033340

[10] Suzuki MM Bird A. DNA methylation landscapes: provocative insights from epigenomics. Nat Rev Genet. (2008) 9:465–76. 10.1038/nrg2341 18463664

[11] Roignant JY Soller MJY. m6A in mRNA: an ancient mechanism for fine-tuning gene expression. Trends Genet. (2017) 33:380–90. 10.1016/j.tig.2017.04.003 28499622

[12] Zhu L Zhong J Jia X. Precision methylome characterization of Mycobacterium tuberculosis complex (MTBC) using PacBio single-molecule real-time (SMRT) technology. Nucleic Acids Res. (2016) 44:730–43. 10.1093/nar/gkv1498 26704977 PMC4737169

[13] Shell SS Prestwich EG Baek SH. DNA methylation impacts gene expression and ensures hypoxic survival of Mycobacterium tuberculosis. PLoS Pathog. (2013) 9:e1003419. 10.1371/journal.ppat.1003419 23853579 PMC3701705

[14] Jiang S Xie Y He Z Zhang Y Zhao Y Chen L. m6ASNP: a tool for annotating genetic variants by m6A function. Gigascience. (2018) 7:35. 10.1093/gigascience/giy035 29617790 PMC6007280

[15] Zhao H Jiang J Wang M Xuan Z. Genome-Wide identification of m6A-associated single-nucleotide polymorphisms in colorectal cancer. Pharmgenomics Pers Med. (2021) 14:887–92. 10.2147/PGPM.S314373 34305406 PMC8297552

[16] He J Yuan L Lin H Lin A Chen H Luo A. Genetic variants in mA modification core genes are associated with glioma risk in Chinese children. Mol Ther Oncolytics. (2021) 20:199–208. 10.1016/j.omto.2020.12.013 33665358 PMC7889446

[17] Lan Q Liu PY Haase J Bell JL Huttelmaier S Liu T. The critical role of RNA m(6)A methylation in cancer. Cancer Res. (2019) 79:1285–92. 10.1158/0008-5472.CAN-18-2965 30894375

[18] Chen XY Zhang J Zhu JS. The role of m(6)A RNA methylation in human cancer. Mol Cancer. (2019) 18:103. 10.1186/s12943-019-1033-z 31142332 PMC6540575

[19] Liu J Cheng J Li L Li Y Zhou H Zhang J. YTHDF1 gene polymorphisms and neuroblastoma susceptibility in Chinese children: an eight-center case-control study. J Cancer. (2021) 12:2465–71. 10.7150/jca.54496 33758623 PMC7974895

[20] Li Z Zhang Z He Z Tang W Li T Zeng Z. A partition-ligation-combination-subdivision EM algorithm for haplotype inference with multiallelic markers: update of the SHEsis (http://analysisbio-xcn). Cell Res. (2009) 19:519–23. 10.1038/cr.2009.33 19290020

[21] Ke S Alemu EA Mertens C Gantman EC Fak JJ Mele A. A majority of m6A residues are in the last exons, allowing the potential for 3' UTR regulation. Genes Dev. (2015) 29:2037–53. 10.1101/gad.269415.115 26404942 PMC4604345

[22] Meyer KD Patil DP Zhou J Zinoviev A Skabkin MA Elemento O. 5' UTR m(6)A promotes cap-independent translation. Cell. (2015) 163:999–1010. 10.1016/j.cell.2015.10.012 26593424 PMC4695625

[23] Ying P Li Y Yang N Wang X Wang H He H. Identification of genetic variants in mA modification genes associated with pancreatic cancer risk in the Chinese population. Arch Toxicol. (2021) 95:1117–28. 10.1007/s00204-021-02978-5 33474615

[24] Feng Y Wang F Pan H Qiu S Lü J Wu L. Obesity-associated gene FTO rs9939609 polymorphism in relation to the risk of tuberculosis. BMC Infect Dis. (2014) 14:592. 10.1186/s12879-014-0592-2 25377722 PMC4226896

[25] Naderi M Hashemi M Dejkam N Bahari G Rezaei M Taheri M. Association study of the FTO gene polymorphisms with the risk of pulmonary tuberculosis in a sample of Iranian population. Acta Microbiol Immunol Hung. (2017) 64:91–9. 10.1556/030.64.2017.010 28357924

[26] Liu Y Lin H Hua RX Zhang J Cheng J Li S. Impact of YTHDF1 gene polymorphisms on Wilms tumor susceptibility: a five-center case-control study. J Clin Lab Anal. (2021) 35:e23875. 10.1002/jcla.23875 34151473 PMC8373325

[27] Zeng H Li M Liu J Zhu J Cheng J Li Y. YTHDF2 gene rs3738067 A>G polymorphism decreases neuroblastoma risk in Chinese children: evidence from an eight-center case-control study. Front Med (Lausanne). (2021) 8:797195. 10.3389/fmed.2021.797195 34970571 PMC8712649

[28] Song J Liu T Zhao Z Hu X Wu Q Peng W. Genetic polymorphisms of long noncoding RNA RP11-37B2. 1 associate with susceptibility of tuberculosis and adverse events of antituberculosis drugs in west China. J Clin Lab Anal. (2019) 33:e22880. 10.1002/jcla.22880 30924187 PMC6595342

[29] Li Y Lu T Wang J Zhuo Z Miao L Yang Z. YTHDC1 gene polymorphisms and neuroblastoma susceptibility in Chinese children. Aging (Albany NY). (2021) 13:25426–39. 10.18632/aging.203760 34897032 PMC8714171

[30] Chen H Li Y Li L Zhu J Yang Z Zhang J. YTHDC1 gene polymorphisms and hepatoblastoma susceptibility in Chinese children: A seven-center case-control study. J Gene Med. (2020) 22:e3249. 10.1002/jgm.3249 32729171

[31] Yang N Ying P Tian J Wang X Mei S Zou D. Genetic variants in m6A modification genes are associated with esophageal squamous-cell carcinoma in the Chinese population. Carcinogenesis. (2020) 41:761–8. 10.1093/carcin/bgaa012 32047883

[32] Chen LZ He CY Su X Peng JL Chen DL Ye Z. SPP1 rs4754 and its epistatic interactions with SPARC polymorphisms in gastric cancer susceptibility. Gene. (2018) 640:43–50. 10.1016/j.gene.2017.09.053 28962925

[33] Bai Y Yang C Wu R Huang L Song S Li W. YTHDF1 regulates tumorigenicity and cancer stem cell-like activity in human colorectal carcinoma. Front Oncol. (2019) 9:332. 10.3389/fonc.2019.00332 31131257 PMC6509179

[34] Zhao X Chen Y Mao Q Jiang X Jiang W Chen J. Overexpression of YTHDF1 is associated with poor prognosis in patients with hepatocellular carcinoma. Cancer Biomark. (2018) 21:859–68. 10.3233/CBM-170791 29439311 PMC13078334

[35] Liu L Liu X Dong Z Li J Yu Y Chen X. N6-methyladenosine-related genomic targets are altered in breast cancer tissue and associated with poor survival. J Cancer. (2019) 10:5447–59. 10.7150/jca.35053 31632489 PMC6775703

[36] Luo Q Rao J Zhang L Fu B Guo Y Huang Z. The study of METTL14, ALKBH5, and YTHDF2 in peripheral blood mononuclear cells from systemic lupus erythematosus. Mol Genet Genomic Med. (2020) 8:e1298. 10.1002/mgg3.1298 32583611 PMC7507441

[37] Luo Q Gao Y Zhang L Rao J Guo Y Huang Z. Decreased ALKBH5, FTO, and YTHDF2 in peripheral blood are as risk factors for rheumatoid arthritis. Biomed Res Int. (2020) 2020:5735279. 10.1155/2020/5735279 32884942 PMC7455827

