# Supplementary material for: Association of N6-methyladenosine readers' genes variation and expression level with pulmonary tuberculosis
Source: Front Public Health. 2022 Aug 22;10:925303. doi: 10.3389/fpubh.2022.925303 (PMC9441624; doi:10.3389/fpubh.2022.925303)
Supplement: Supplementary file 1 [file Table_1.DOC]

**Table S1** The demographic and clinical characteristics of PTB patients and normal controls

| Characteristics | PTB patients | Normal controls |
| --- | --- | --- |
| Genotyping experiment | | |
| Demographic characteristics |  |  |
| Age (years) | 45.42 ± 17.74 | 43.43 ± 12.95 |
| Sex (male/female) | 264/193 | 202/264 |
| Clinical characteristics |  |  |
| Fever [n (%)] | 71 (15.54) | NA |
| Drug resistance [n (%)] | 73 (15.97) | NA |
| DILI [n (%)] | 66 (14.44) | NA |
| Pulmonary infection [n (%)] | 81 (17.72) | NA |
| Hypoproteinemia [n (%)] | 39 (8.53) | NA |
| Leukopenia [n (%)] | 31 (6.78) | NA |
| Sputum smear-positive [n (%)] | 125 (27.35) | NA |
| PCR experiment | | |
| Demographic characteristics |  |  |
| Age (years) | 49.83 ± 18.59 | 48.47 ± 17.40 |
| Sex (male/female) | 51/27 | 57/29 |
| Clinical characteristics |  |  |
| Fever [n (%)] | 15 (19.23) | NA |
| Drug resistance [n (%)] | 5 (6.41) | NA |
| DILI [n (%)] | 8 (10.26) | NA |
| Pulmonary infection [n (%)] | 11 (14.10) | NA |
| Hypoproteinemia [n (%)] | 16 (20.51) | NA |
| Leukopenia [n (%)] | 6 (7.69) | NA |
| Sputum smear-positive [n (%)] | 27 (34.61) | NA |

PTB, pulmonary tuberculosis; DILI: drug-induced liver injury; NA: not applicable; ^a^part of the study subjects of data missing.
