# Supplementary material for: Association of N6-methyladenosine readers' genes variation and expression level with pulmonary tuberculosis
Source: Front Public Health. 2022 Aug 22;10:925303. doi: 10.3389/fpubh.2022.925303 (PMC9441624; doi:10.3389/fpubh.2022.925303)
Supplement: Supplementary file 2 [file Table_2.DOC]

**Table S2** The association between m6A readers’ genes polymorphisms and clinical features of PTB patients

| SNP | Allele | Clinical features | Group | Genotype | | | P value | Allele | | P value |
| --- | --- | --- | --- | --- | --- | --- | --- | --- | --- | --- |
|  | (M/m) |  |  | MM | Mm | mm |  | M | m |  |
| YTHDF1 | | | | | | | | | | |
| rs6122103 | G/A | fever | + | 37 (52.11) | 28 (39.44) | 6 (8.45) | 0.536 | 102 (71.83) | 40 (28.17) | 0.255 |
|  |  |  | - | 177 (45.85) | 163 (42.23) | 46 (11.92) |  | 517 (66.97) | 255 (33.03) |  |
|  |  | drug resistance | + | 36 (49.32) | 28 (38.36) | 9 (12.33) | 0.807 | 100 (68.49) | 46 (31.51) | 0.828 |
|  |  |  | - | 178 (46.35) | 163 (42.45) | 43 (11.2) |  | 519 (67.58) | 249 (32.42) |  |
|  |  | DILI | + | 30 (45.45) | 28 (42.42) | 8 (12.12) | 0.963 | 88 (66.67) | 44 (33.33) | 0.779 |
|  |  |  | - | 184 (47.06) | 163 (41.69) | 44 (11.25) |  | 531 (67.9) | 251 (32.1) |  |
|  |  | pulmonary infection | + | 45 (55.56) | 27 (33.33) | 9 (11.11) | 0.193 | 117 (72.22) | 45 (27.78) | 0.177 |
|  |  |  | - | 169 (44.95) | 164 (43.62) | 43 (11.44) |  | 502 (66.76) | 250 (33.24) |  |
|  |  | hypoproteinemia | + | 21 (53.85) | 13 (33.33) | 5 (12.82) | 0.533 | 55 (70.51) | 23 (29.49) | 0.582 |
|  |  |  | - | 193 (46.17) | 178 (42.58) | 47 (11.24) |  | 564 (67.46) | 272 (32.54) |  |
|  |  | leukopenia | + | 13 (41.94) | 13 (41.94) | 5 (16.13) | 0.660 | 39 (62.90) | 23 (37.10) | 0.400 |
|  |  |  | - | 201 (47.18) | 178 (41.78) | 47 (11.03) |  | 580 (68.08) | 272 (31.92) |  |
|  |  | sputum smear | + | 67 (44.08) | 71 (46.71) | 14 (9.21) | 0.499 | 205 (67.43) | 99 (32.57) | 0.534 |
|  |  |  | - | 150 (43.60) | 150 (43.60) | 44 (12.79) |  | 450 (65.41) | 238 (34.59) |  |
| rs6011668 | C/T | fever | + | 53 (74.65) | 16 (22.54) | 2 (2.82) | 0.381 | 122 (85.92) | 20 (14.08) | 0.630 |
|  |  |  | - | 270 (69.95) | 111 (28.76) | 5 (1.30) |  | 651 (84.33) | 121 (15.67) |  |
|  |  | drug resistance | + | 46 (63.01) | 26 (35.62) | 1 (1.37) | 0.266 | 118 (80.82) | 28 (19.18) | 0.171 |
|  |  |  | - | 277 (72.14) | 101 (26.3) | 6 (1.56) |  | 655 (85.29) | 113 (14.71) |  |
|  |  | DILI | + | 45 (68.18) | 20 (30.30) | 1 (1.52) | 0.885 | 110 (83.33) | 22 (16.67) | 0.670 |
|  |  |  | - | 278 (71.10) | 107 (27.37) | 6 (1.53) |  | 663 (84.78) | 119 (15.22) |  |
|  |  | pulmonary infection | + | 56 (69.14) | 23 (28.40) | 2 (2.47) | 0.737 | 135 (83.33) | 27 (16.67) | 0.630 |
|  |  |  | - | 267 (71.01) | 104 (27.66) | 5 (1.33) |  | 638 (84.84) | 114 (15.16) |  |
|  |  | hypoproteinemia | + | 29 (74.36) | 8 (20.51) | 2 (5.13) | 0.106 | 66 (84.62) | 12 (15.38) | 0.991 |
|  |  |  | - | 294 (70.33) | 119 (28.47) | 5 (1.20) |  | 707 (84.57) | 129 (15.43) |  |
|  |  | leukopenia | + | 22 (70.97) | 8 (25.81) | 1 (3.23) | 0.715 | 52 (83.87) | 10 (16.13) | 0.874 |
|  |  |  | - | 301 (70.66) | 119 (27.93) | 6 (1.41) |  | 721 (84.62) | 131 (15.38) |  |
|  |  | sputum smear | + | 109 (71.71) | 40 (26.32) | 3 (1.97) | 0.742 | 258 (84.87) | 46 (15.13) | 0.995 |
|  |  |  | - | 244 (70.93) | 96 (27.91) | 4 (1.16) |  | 584 (84.88) | 104 (15.12) |  |
| YTHDF2 | | | | | | | | | | |
| rs602345 | C/T | fever | + | 50 (70.42) | 20 (28.17) | 1 (1.41) | 0.492 | 120 (84.51) | 22 (15.49) | 0.865 |
|  |  |  | - | 277 (71.76) | 94 (24.35) | 15 (3.89) |  | 648 (83.94) | 124 (16.06) |  |
|  |  | drug resistance | + | 55 (70.51) | 19 (24.36) | 4 (5.13) | 0.711 | 129 (82.69) | 27 (17.31) | 0.527 |
|  |  |  | - | 332 (72.65) | 110 (24.07) | 15 (3.28) |  | 774 (84.68) | 140 (15.32) |  |
|  |  | DILI | + | 52 (70.27) | 19 (25.68) | 3 (4.05) | 0.905 | 123 (83.11) | 25 (16.89) | 0.643 |
|  |  |  | - | 335 (72.67) | 110 (23.86) | 16 (3.47) |  | 780 (84.60) | 142 (15.40) |  |
|  |  | pulmonary infection | + | 64 (69.57) | 25 (27.17) | 3 (3.26) | 0.750 | 153 (83.15) | 31 (16.85) | 0.610 |
|  |  |  | - | 323 (72.91) | 104 (23.48) | 16 (3.61) |  | 750 (84.65) | 136 (15.35) |  |
|  |  | hypoproteinemia | + | 42 (76.36) | 12 (21.82) | 1 (1.82) | 0.674 | 96 (87.27) | 14 (12.73) | 0.380 |
|  |  |  | - | 345 (71.88) | 117 (24.38) | 18 (3.75) |  | 807 (84.06) | 153 (15.94) |  |
|  |  | leukopenia | + | 27 (72.97) | 10 (27.03) | 0 (0) | 0.460 | 64 (86.49) | 10 (13.51) | 0.607 |
|  |  |  | - | 360 (72.29) | 119 (23.90) | 19 (3.82) |  | 839 (84.24) | 157 (15.76) |  |
|  |  | sputum smear | + | 111 (73.03) | 38 (25.00) | 3 (1.97) | 0.568 | 260 (85.53) | 44 (14.47) | 0.622 |
|  |  |  | - | 249 (72.38) | 82 (23.84) | 13 (3.78) |  | 580 (84.30) | 108 (15.70) |  |
| rs3738067 | A/G | fever | + | 37 (52.11) | 24 (33.80) | 10 (14.08) | 0.132 | 98 (69.01) | 44 (30.99) | 0.164 |
|  |  |  | - | 217 (56.22) | 142 (36.79) | 27 (6.99) |  | 576 (74.61) | 196 (25.39) |  |
|  |  | drug resistance | + | 41 (52.56) | 32 (41.03) | 5 (6.41) | 0.416 | 114 (73.08) | 42 (26.92) | 0.727 |
|  |  |  | - | 263 (57.55) | 154 (33.7) | 40 (8.75) |  | 680 (74.40) | 234 (25.60) |  |
|  |  | DILI | + | 41 (55.41) | 25 (33.78) | 8 (10.81) | 0.725 | 107 (72.30) | 41 (27.70) | 0.568 |
|  |  |  | - | 263 (57.05) | 161 (34.92) | 37 (8.03) |  | 687 (74.51) | 235 (25.49) |  |
|  |  | pulmonary infection | + | 49 (53.26) | 31 (33.7) | 12 (13.04) | 0.210 | 129 (70.11) | 55 (29.89) | 0.163 |
|  |  |  | - | 255 (57.56) | 155 (34.99) | 33 (7.45) |  | 665 (75.06) | 221 (24.94) |  |
|  |  | hypoproteinemia | + | 39 (70.91) | 9 (16.36) | 7 (12.73) | 0.009 | 87 (79.09) | 23 (20.91) | 0.216 |
|  |  |  | - | 265 (55.21) | 177 (36.88) | 38 (7.92) |  | 707 (73.65) | 253 (26.35) |  |
|  |  | leukopenia | + | 22 (59.46) | 12 (32.43) | 3 (8.11) | 0.944 | 56 (75.68) | 18 (24.32) | 0.764 |
|  |  |  | - | 282 (56.63) | 174 (34.94) | 42 (8.43) |  | 738 (74.10) | 258 (25.90) |  |
|  |  | sputum smear | + | 88 (57.89) | 51 (33.55) | 13 (8.55) | 0.829 | 227 (74.67) | 77 (25.33) | 0.819 |
|  |  |  | - | 192 (55.81) | 125 (36.34) | 27 (7.85) |  | 509 (73.98) | 179 (26.02) |  |
| YTHDF3 | | | | | | | | | | |
| rs7464 | A/G | fever | + | 38 (53.52) | 26 (36.62) | 7 (9.86) | 0.585 | 102 (71.83) | 40 (28.17) | 0.573 |
|  |  |  | - | 211 (54.66) | 150 (38.86) | 25 (6.48) |  | 572 (74.09) | 200 (25.91) |  |
|  |  | drug resistance | + | 40 (51.28) | 32 (41.03) | 6 (7.69) | 0.981 | 112 (71.79) | 44 (28.21) | 0.848 |
|  |  |  | - | 239 (52.3) | 185 (40.48) | 33 (7.22) |  | 663 (72.54) | 251 (27.46) |  |
|  |  | DILI | + | 43 (58.11) | 27 (36.49) | 4 (5.41) | 0.508 | 113 (76.35) | 35 (23.65) | 0.250 |
|  |  |  | - | 236 (51.19) | 190 (41.21) | 35 (7.59) |  | 662 (71.80) | 260 (28.20) |  |
|  |  | pulmonary infection | + | 50 (54.35) | 39 (42.39) | 3 (3.26) | 0.263 | 139 (75.54) | 45 (24.46) | 0.299 |
|  |  |  | - | 229 (51.69) | 178 (40.18) | 36 (8.13) |  | 636 (71.78) | 250 (28.22) |  |
|  |  | hypoproteinemia | + | 31 (56.36) | 21 (38.18) | 3 (5.45) | 0.749 | 83 (75.45) | 27 (24.55) | 0.454 |
|  |  |  | - | 248 (51.67) | 196 (40.83) | 36 (7.50) |  | 692 (72.08) | 268 (27.92) |  |
|  |  | leukopenia | + | 14 (37.84) | 20 (54.05) | 3 (8.11) | 0.184 | 48 (64.86) | 26 (35.14) | 0.131 |
|  |  |  | - | 265 (53.21) | 197 (39.56) | 36 (7.23) |  | 727 (72.99) | 269 (27.01) |  |
|  |  | sputum smear | + | 76 (50.00) | 65 (42.76) | 11 (7.24) | 0.863 | 217 (71.38) | 87 (28.62) | 0.929 |
|  |  |  | - | 177 (51.45) | 139 (40.41) | 28 (8.14) |  | 493 (71.66) | 195 (28.34) |  |
| rs12549833 | A/G | fever | + | 34 (47.89) | 30 (42.25) | 7 (9.86) | 0.660 | 98 (69.01) | 44 (30.99) | 0.358 |
|  |  |  | - | 165 (42.75) | 172 (44.56) | 49 (12.69) |  | 502 (65.03) | 270 (34.97) |  |
|  |  | drug resistance | + | 38 (48.72) | 33 (42.31) | 7 (8.97) | 0.506 | 109 (69.87) | 47 (30.13) | 0.245 |
|  |  |  | - | 193 (42.23) | 209 (45.73) | 55 (12.04) |  | 595 (65.10) | 319 (34.90) |  |
|  |  | DILI | + | 30 (40.54) | 33 (44.59) | 11 (14.86) | 0.625 | 93 (62.84) | 55 (37.16) | 0.414 |
|  |  |  | - | 201 (43.6) | 209 (45.34) | 51 (11.06) |  | 611 (66.27) | 311 (33.73) |  |
|  |  | pulmonary infection | + | 42 (45.65) | 38 (41.30) | 12 (13.04) | 0.691 | 122 (66.30) | 62 (33.70) | 0.873 |
|  |  |  | - | 189 (42.66) | 204 (46.05) | 50 (11.29) |  | 582 (65.69) | 304 (34.31) |  |
|  |  | hypoproteinemia | + | 20 (36.36) | 25 (45.45) | 10 (18.18) | 0.228 | 65 (59.09) | 45 (40.91) | 0.118 |
|  |  |  | - | 211 (43.96) | 217 (45.21) | 52 (10.83) |  | 639 (66.56) | 321 (33.44) |  |
|  |  | leukopenia | + | 15 (40.54) | 16 (43.24) | 6 (16.22) | 0.659 | 46 (62.16) | 28 (37.84) | 0.495 |
|  |  |  | - | 216 (43.37) | 226 (45.38) | 56 (11.24) |  | 658 (66.06) | 338 (33.94) |  |
|  |  | sputum smear | + | 61 (40.13) | 76 (50.00) | 15 (9.87) | 0.261 | 198 (65.13) | 106 (34.87) | 0.627 |
|  |  |  | - | 157 (45.64) | 145 (42.15) | 42 (12.21) |  | 459 (66.72) | 229 (33.28) |  |
| YTHDC1 | | | | | | | | | | |
| rs3813832 | T/C | fever | + | 27 (38.03) | 38 (53.52) | 6 (8.45) | 0.044 | 92 (64.79) | 50 (35.21) | 0.027 |
|  |  |  | - | 209 (54.15) | 152 (39.38) | 25 (6.48) |  | 570 (73.83) | 202 (26.17) |  |
|  |  | drug resistance | + | 48 (61.54) | 25 (32.05) | 5 (6.41) | 0.088 | 121 (77.56) | 35 (22.44) | 0.082 |
|  |  |  | - | 221 (48.36) | 205 (44.86) | 31 (6.78) |  | 647 (70.79) | 267 (29.21) |  |
|  |  | DILI | + | 29 (39.19) | 41 (55.41) | 4 (5.41) | 0.067 | 99 (66.89) | 49 (33.11) | 0.155 |
|  |  |  | - | 240 (52.06) | 189 (41.00) | 32 (6.94) |  | 669 (72.56) | 253 (27.44) |  |
|  |  | pulmonary infection | + | 48 (52.17) | 41 (44.57) | 3 (3.26) | 0.345 | 137 (74.46) | 47 (25.54) | 0.375 |
|  |  |  | - | 221 (49.89) | 189 (42.66) | 33 (7.45) |  | 631 (71.22) | 255 (28.78) |  |
|  |  | hypoproteinemia | + | 27 (49.09) | 27 (49.09) | 1 (1.82) | 0.253 | 81 (73.64) | 29 (26.36) | 0.647 |
|  |  |  | - | 242 (50.42) | 203 (42.29) | 35 (7.29) |  | 687 (71.56) | 273 (28.44) |  |
|  |  | leukopenia | + | 17 (45.95) | 19 (51.35) | 1 (2.70) | 0.416 | 53 (71.62) | 21 (28.38) | 0.976 |
|  |  |  | - | 252 (50.60) | 211 (42.37) | 35 (7.03) |  | 715 (71.79) | 281 (28.21) |  |
|  |  | sputum smear | + | 82 (53.95) | 60 (39.47) | 10 (6.58) | 0.718 | 224 (73.68) | 80 (26.32) | 0.526 |
|  |  |  | - | 172 (50.00) | 148 (43.02) | 24 (6.98) |  | 492 (71.51) | 196 (28.49) |  |
| rs17592288 | A/C | fever | + | 65 (91.55) | 6 (8.45) | 0 (0) | 0.911 | 136 (95.77) | 6 (4.23) | 0.870 |
|  |  |  | - | 352 (91.19) | 33 (8.55) | 1 (0.26) |  | 737 (95.47) | 35 (4.53) |  |
|  |  | drug resistance | + | 75 (96.15) | 3 (3.85) | 0 (0) | 0.308 | 153 (98.08) | 3 (1.92) | 0.124 |
|  |  |  | - | 416 (91.03) | 40 (8.75) | 1 (0.22) |  | 872 (95.40) | 42 (4.60) |  |
|  |  | DILI | + | 70 (94.59) | 4 (5.41) | 0 (0) | 0.614 | 144 (97.30) | 4 (2.70) | 0.326 |
|  |  |  | - | 421 (91.32) | 39 (8.46) | 1 (0.22) |  | 881 (95.55) | 41 (4.45) |  |
|  |  | pulmonary infection | + | 85 (92.39) | 6 (6.52) | 1 (1.09) | 0.077 | 176 (95.65) | 8 (4.35) | 0.916 |
|  |  |  | - | 406 (91.65) | 37 (8.35) | 0 (0) |  | 849 (95.82) | 37 (4.18) |  |
|  |  | hypoproteinemia | + | 47 (85.45) | 8 (14.55) | 0 (0) | 0.164 | 102 (92.73) | 8 (7.27) | 0.091 |
|  |  |  | - | 444 (92.5) | 35 (7.29) | 1 (0.21) |  | 923 (96.15) | 37 (3.85) |  |
|  |  | leukopenia | + | 34 (91.89) | 3 (8.11) | 0 (0) | 0.963 | 71 (95.95) | 3 (4.05) | 0.946 |
|  |  |  | - | 457 (91.77) | 40 (8.03) | 1 (0.20) |  | 954 (95.78) | 42 (4.22) |  |
|  |  | sputum smear | + | 139 (91.45) | 12 (7.89) | 1 (0.66) | 0.321 | 290 (95.39) | 14 (4.61) | 0.699 |
|  |  |  | - | 316 (91.86) | 28 (8.14) | 0 (0) |  | 660 (95.93) | 28 (4.07) |  |
| rs2293596 | T/C | fever | + | 45 (63.38) | 24 (33.80) | 2 (2.82) | 0.673 | 114 (80.28) | 28 (19.72) | 0.879 |
|  |  |  | - | 255 (66.06) | 114 (29.53) | 17 (4.40) |  | 624 (80.83) | 148 (19.17) |  |
|  |  | drug resistance | + | 49 (62.82) | 25 (32.05) | 4 (5.13) | 0.860 | 123 (78.85) | 33 (21.15) | 0.581 |
|  |  |  | - | 300 (65.65) | 138 (30.20) | 19 (4.16) |  | 738 (80.74) | 176 (19.26) |  |
|  |  | DILI | + | 47 (63.51) | 26 (35.14) | 1 (1.35) | 0.303 | 120 (81.08) | 28 (18.92) | 0.839 |
|  |  |  | - | 302 (65.51) | 137 (29.72) | 22 (4.77) |  | 741 (80.37) | 181 (19.63) |  |
|  |  | pulmonary infection | + | 64 (69.57) | 23 (25.00) | 5 (5.43) | 0.418 | 151 (82.07) | 33 (17.93) | 0.549 |
|  |  |  | - | 285 (64.33) | 140 (31.6) | 18 (4.06) |  | 710 (80.14) | 176 (19.86) |  |
|  |  | hypoproteinemia | + | 37 (67.27) | 17 (30.91) | 1 (1.82) | 0.631 | 91 (82.73) | 19 (17.27) | 0.528 |
|  |  |  | - | 312 (65.00) | 146 (30.42) | 22 (4.58) |  | 770 (80.21) | 190 (19.79) |  |
|  |  | leukopenia | + | 28 (75.68) | 9 (24.32) | 0 (0) | 0.239 | 65 (87.84) | 9 (12.16) | 0.097 |
|  |  |  | - | 321 (64.46) | 154 (30.92) | 23 (4.62) |  | 796 (79.92) | 200 (20.08) |  |
|  |  | sputum smear | + | 98 (64.47) | 48 (31.58) | 6 (3.95) | 0.865 | 244 (80.26) | 60 (19.74) | 0.907 |
|  |  |  | - | 223 (64.83) | 104 (30.23) | 17 (4.94) |  | 550 (79.94) | 138 (20.06) |  |
| YTHDC2 | | | | | | | | | | |
| rs6594732 | C/A | fever | + | 45 (63.38) | 25 (35.21) | 1 (1.41) | 0.328 | 115 (80.99) | 27 (19.01) | 0.977 |
|  |  |  | - | 257 (66.58) | 112 (29.02) | 17 (4.4) |  | 626 (81.09) | 146 (18.91) |  |
|  |  | drug resistance | + | 49 (62.82) | 26 (33.33) | 3 (3.85) | 0.922 | 124 (79.49) | 32 (20.51) | 0.812 |
|  |  |  | - | 296 (64.77) | 142 (31.07) | 19 (4.16) |  | 734 (80.31) | 180 (19.69) |  |
|  |  | DILI | + | 39 (52.70) | 29 (39.19) | 6 (8.11) | 0.033 | 107 (72.30) | 41 (27.70) | 0.008 |
|  |  |  | - | 306 (66.38) | 139 (30.15) | 16 (3.47) |  | 751 (81.45) | 171 (18.55) |  |
|  |  | pulmonary infection | + | 51 (55.43) | 35 (38.04) | 6 (6.52) | 0.103 | 137 (74.46) | 47 (25.54) | 0.032 |
|  |  |  | - | 294 (66.37) | 133 (30.02) | 16 (3.61) |  | 721 (81.38) | 165 (18.62) |  |
|  |  | hypoproteinemia | + | 33 (60.00) | 20 (36.36) | 2 (3.64) | 0.703 | 86 (78.18) | 24 (21.82) | 0.578 |
|  |  |  | - | 312 (65.00) | 148 (30.83) | 20 (4.17) |  | 772 (80.42) | 188 (19.58) |  |
|  |  | leukopenia | + | 18 (48.65) | 17 (45.95) | 2 (5.41) | 0.112 | 53 (71.62) | 21 (28.38) | 0.055 |
|  |  |  | - | 327 (65.66) | 151 (30.32) | 20 (4.02) |  | 805 (80.82) | 191 (19.18) |  |
|  |  | sputum smear | + | 106 (69.74) | 44 (28.95) | 2 (1.32) | 0.039 | 256 (84.21) | 48 (15.79) | 0.020 |
|  |  |  | - | 211 (61.34) | 113 (32.85) | 20 (5.81) |  | 535 (77.76) | 153 (22.24) |  |
| rs2416282 | A/C | fever | + | 15 (21.13) | 45 (63.38) | 11 (15.49) | 0.013 | 75 (52.82) | 67 (47.18) | 0.420 |
|  |  |  | - | 132 (34.20) | 172 (44.56) | 82 (21.24) |  | 436 (56.48) | 336 (43.52) |  |
|  |  | drug resistance | + | 22 (28.21) | 38 (48.72) | 18 (23.08) | 0.674 | 82 (52.56) | 74 (47.44) | 0.379 |
|  |  |  | - | 146 (31.95) | 223 (48.8) | 88 (19.26) |  | 515 (56.35) | 399 (43.65) |  |
|  |  | DILI | + | 20 (27.03) | 33 (44.59) | 21 (28.38) | 0.134 | 73 (49.32) | 75 (50.68) | 0.088 |
|  |  |  | - | 148 (32.10) | 228 (49.46) | 85 (18.44) |  | 524 (56.83) | 398 (43.17) |  |
|  |  | pulmonary infection | + | 25 (27.17) | 43 (46.74) | 24 (26.09) | 0.230 | 93 (50.54) | 91 (49.46) | 0.115 |
|  |  |  | - | 143 (32.28) | 218 (49.21) | 82 (18.51) |  | 504 (56.88) | 382 (43.12) |  |
|  |  | hypoproteinemia | + | 15 (27.27) | 26 (47.27) | 14 (25.45) | 0.510 | 56 (50.91) | 54 (49.09) | 0.276 |
|  |  |  | - | 153 (31.88) | 235 (48.96) | 92 (19.17) |  | 541 (56.35) | 419 (43.65) |  |
|  |  | leukopenia | + | 12 (32.43) | 18 (48.65) | 7 (18.92) | 0.985 | 42 (56.76) | 32 (43.24) | 0.863 |
|  |  |  | - | 156 (31.33) | 243 (48.80) | 99 (19.88) |  | 555 (55.72) | 441 (44.28) |  |
|  |  | sputum smear | + | 46 (30.26) | 69 (45.39) | 37 (24.34) | 0.190 | 161 (52.96) | 143 (47.04) | 0.240 |
|  |  |  | - | 108 (31.40) | 176 (51.16) | 60 (17.44) |  | 392 (56.98) | 296 (43.02) |  |
