# Supplementary material for: Association of N6-methyladenosine readers' genes variation and expression level with pulmonary tuberculosis
Source: Front Public Health. 2022 Aug 22;10:925303. doi: 10.3389/fpubh.2022.925303 (PMC9441624; doi:10.3389/fpubh.2022.925303)
Supplement: Supplementary file 3 [file Table_3.DOC]

| Clinical features | Group | N | YTHDF1 level | P value | YTHDF2 level | P value | YTHDF3 level | P value | YTHDC1 level | P value | YTHDC2 level | P value |
| --- | --- | --- | --- | --- | --- | --- | --- | --- | --- | --- | --- | --- |
| fever | + | 15 | 0.265 (0.214, 0.423) | 0.052 | 0.395 (0.251, 0.821) | 0.326 | 0.357 (0.146, 0.756) | 0.680 | 0.272 (0.191, 0.829) | 0.539 | 0.404 (0.218, 0.104) | 0.617 |
|  | - | 63 | 0.407 (0.237, 0.848) |  | 0.481 (0.267, 0.911) |  | 0.412 (0.201, 0.752) |  | 0.403 (0.248, 0.573) |  | 0.525 (0.321, 0.877) |  |
| drug resistance | + | 5 | 0.851 (0.249, 1.012) | 0.249 | 1.147 (0.278, 1.613) | 0.217 | 0.662 (0.240, 1.130) | 0.343 | 0.442 (0.145, 1.083) | 0.830 | 1.134 (0.210, 2.780) | 0.313 |
|  | - | 73 | 0.356 (0.231, 0.673) |  | 0.475 (0.265, 0.831) |  | 0.411 (0.197, 0.707) |  | 0.367 (0.214, 0.596) |  | 0.497 (0.304, 0.863) |  |
| DILI | + | 8 | 0.588 (0.294, 0.957) | 0.138 | 0.556 (0.304, 0.893) | 0.633 | 0.461 (0.185, 0.981) | 0.717 | 0.375 (0.209, 0.759) | 0.947 | 0.674 (0.311, 0.965) | 0.779 |
|  | - | 70 | 0.355 (0.227, 0.668) |  | 0.471 (0.261, 0.910) |  | 0.411 (0.198, 0.753) |  | 0.367 (0.215, 0.585) |  | 0.479 (0.301, 0.885) |  |
| pulmonary infection | + | 11 | 0.263 (0.122, 0.354) | 0.089 | 0.458 (0.268, 0.842) | 0.590 | 0.423 (0.193, 0.620) | 0.869 | 0.309 (0.195, 0.553) | 0.495 | 0.422 (0.307, 0.859) | 0.600 |
|  | - | 67 | 0.814 (0.618, 1.161) |  | 0.916 (0.629, 1.437) |  | 0.771 (0.409, 1.299) |  | 0.875 (0.459, 1.249) |  | 0.927 (0.543, 1.417) |  |
| hypoproteinemia | + | 16 | 0.272 (0.145, 0.765) | 0.304 | 0.429 (0.214, 0.929) | 0.701 | 0.429 (0.195, 0.965) | 0.748 | 0.364 (0.192, 0.867) | 0.941 | 0.544 (0.253, 0.854) | 0.512 |
|  | - | 62 | 0.394 (0.255, 0.721) |  | 0.479 (0.273, 0.842) |  | 0.412 (0.198, 0.753) |  | 0.367 (0.223, 0.619) |  | 0.479 (0.318.0.933) |  |
| leukopenia | + | 6 | 0.475 (0.252, 0.662) | 0.680 | 0.519 (0.372, 0.806) | 0.736 | 0.819 (0.338, 1.101) | 0.171 | 0.839 (0.301, 1.310) | 0.139 | 0.892 (0.165, 1.120) | 0.680 |
|  | - | 72 | 0.355 (0.230, 0.727) |  | 0.471 (0.265, 0.910) |  | 0.409 (0.196, 0.662) |  | 0.361 (0.212, 0.571) |  | 0.479 (0.307, 0.864) |  |
| sputum smear-positive | + | 27 | 0.354 (0.207, 0.823) | 0.797 | 0.515 (0.264, 0.926) | 0.757 | 0.412 (0.212, 0.752) | 0.937 | 0.357 (0.195, 0.620) | 0.979 | 0.596 (0.307, 1.037) | 0.539 |
|  | - | 51 | 0.383 (0.237, 0.717) |  | 0.466 (0.275, 0.820) |  | 0.412 (0.196, 0.756) |  | 0.368 (0.217, 0.573) |  | 0.456 (0.302, 0.856) |  |

**Table S3** The association between m6A readers’ genes expression levels and several clinical features in PTB patients

+/-: with/without; median (interquartile range); ^a^part of the study subjects of data missing.
