# Supplementary material for: Association of N6-methyladenosine readers' genes variation and expression level with pulmonary tuberculosis
Source: Front Public Health. 2022 Aug 22;10:925303. doi: 10.3389/fpubh.2022.925303 (PMC9441624; doi:10.3389/fpubh.2022.925303)
Supplement: Supplementary file 4 [file Table_4.DOC]

**Table S4** Association between m6A readers’ genes polymorphisms with

their expression levels in PTB patients

| YTHDF1 SNP | Genotype | number | YTHDF1 level | P value |
| --- | --- | --- | --- | --- |
| rs6122103 | AA | 4 | 0.672 (0.322, 0.926) | 0.209 |
|  | GA | 25 | 0.270 (0.186, 0.634) |  |
|  | GG | 33 | 0.378 (0.241, 0.791) |  |
| rs6011668 | TT | 0 | 0 | 0.210 |
|  | TC | 17 | 0.383 (0.281, 0.969) |  |
|  | CC | 45 | 0.314 (0.210, 0.700) |  |
| YTHDF2 SNP | Genotype | number | YTHDF2 level | P value |
| rs602345 | TT | 2 | 0.760 (0.210, 1.310) | 0.629 |
|  | TC | 21 | 0.444 (0.234, 0.642) |  |
|  | CC | 39 | 0.481 (0.275, 0.935) |  |
| rs3738067 | GG | 2 | 0.760 (0.210, 1.310) | 0.682 |
|  | GA | 27 | 0.444 (0.239, 0.672) |  |
|  | AA | 33 | 0.481 (0.273, 1.055) |  |
| YTHDF3 SNP | Genotype | number | YTHDF3 level | P value |
| rs7464 | GG | 4 | 0.669 (0.454, 1.783) | 0.158 |
|  | GA | 34 | 0.305 (0.188, 0.614) |  |
|  | AA | 24 | 0.394 (0.139, 0.740) |  |
| rs12549833 | GG | 5 | 0.092 (0.076, 0.702) | 0.312 |
|  | AG | 28 | 0.319 (0.179, 0.763) |  |
|  | AA | 29 | 0.411 (0.202, 0.630) |  |
| YTHDC1 SNP | Genotype | number | YTHDC1 level | P value |
| rs3813832 | CC | 1 | 0.019 | 0.101 |
|  | TC | 25 | 0.314 (0.160, 0.529) |  |
|  | TT | 36 | 0.405 (0.225, 0.780) |  |
| rs17592288 | CC | 0 |  | 0.312 |
|  | AC | 7 | 0.567 (0.264, 0.819) |  |
|  | AA | 55 | 0.321 (0.190, 0.533) |  |
| rs2293596 | CC | 2 | 0.422 (0.314, 0.530) | 0.357 |
|  | TC | 21 | 0.394 (0.241, 0.773) |  |
|  | TT | 39 | 0.312 (0.160, 0.533) |  |
| YTHDC2 SNP | Genotype | number | YTHDC2 level | P value |
| rs6594732 | AA | 2 | 3.124 (1.130, 5.119) | 0.106 |
|  | CA | 18 | 0.383 (0.191, 0.894) |  |
|  | CC | 42 | 0.477 (0.288, 0.854) |  |
| rs2416282 | CC | 10 | 0.595 (0.317, 1.032) | 0.740 |
|  | CA | 30 | 0.475 (0.261, 0.857) |  |
|  | AA | 22 | 0.429 (0.215, 0.942) |  |

M

Median (interquartile range)
